# Circulating Hepcidin Levels Are an Independent Predictor of Survival in Microsatellite Stable Metastatic Colorectal Cancer Patient Candidates for Standard First-Line Treatment

**DOI:** 10.3390/cancers16233977

**Published:** 2024-11-27

**Authors:** Vincenzo Formica, Antonio Di Grazia, Maria Vittoria Bonomo, Rachele Frascatani, Roberto Mancone, Giovanni Monteleone

**Affiliations:** 1 Medical Oncology Unit, Fondazione Policlinico “Tor Vergata”, 00133 Rome, Italy; v.formica1@gmail.com (V.F.); mariavittoriabonomo95@gmail.com (M.V.B.); 2 Department of Systems Medicine, University of Rome “Tor Vergata”, 00133 Rome, Italy; adigrazia2000@yahoo.it (A.D.G.); rakfrasc@gmail.com (R.F.); 3 Gastroenterology Unit, Fondazione Policlinico “Tor Vergata”, 00133 Rome, Italy; roberto.mancone@yahoo.it

**Keywords:** colon carcinogenesis, iron metabolism, FOLFOX, RAS/BRAF mutations

## Abstract

Background & Aim. Hepcidin, a key hormone in iron homeostasis, is synthesized by colorectal cancer (CRC) cells, particularly in the late stages of tumorigenesis. This study aimed to ascertain whether the serum levels of hepcidin could serve as a prognostic biomarker in microsatellite stable (MSS) metastatic CRC (mCRC). Specifically, we assessed the predictive value of baseline serum hepcidin levels for the overall survival (OS) of patients with MSS mCRC receiving first-line treatment with FOLFOX-panitumumab (RAS/BRAF wild-type) or FOLFOX-bevacizumab (RAS or BRAF mutations). Methods. Serum samples were prospectively collected from 35 normal healthy volunteers (normal controls) and 55 patients with MSS mCRC and analyzed for their content of hepcidin by ELISA. Results. Serum hepcidin levels were significantly greater in patients with mCRC than in the normal controls. In the mCRC group, patients with baseline levels of hepcidin greater than 40 ng/mL had a significantly shorter 1-year OS rate (39%) than those with hepcidin levels lower than 40 ng/mL (80%) [hazard ratio (HR): 2.94; 95% CI: 1.27–6.84; *p* = 0.01]. A multivariate Cox regression analysis showed that the pre-treatment serum hepcidin levels were an independent prognostic factor for OS, not influenced by other well-known prognostic factors (i.e., CEA status, Karnofsky performance score, number of metastatic sites, RAS/BRAF mutations), and this was evident across all major patient subgroups. Conclusions. Our data show that baseline serum levels of hepcidin are an independent risk factor for OS in MSS mCRC patients undergoing standard first-line treatment. Further prospective and extensive studies are needed to confirm and validate our findings.

## Simple Summary

Hepcidin, a peptide hormone, is overexpressed in colorectal cancer, mainly in the advanced stages. This study assessed whether the pre-treatment serum levels of hepcidin are a predictive factor of the overall survival of patients with microsatellite stable metastatic colorectal cancer receiving first-line treatment with FOLFOX-panitumumab (RAS/BRAF wild-type) or FOLFOX-bevacizumab (RAS or BRAF mutations). Our data showed that patients with metastatic colorectal cancer had higher serum levels of hepcidin than the controls, and patients with circulating levels of hepcidin greater than 40 ng/mL had a reduced overall survival as compared to those with values lower than 40 ng/mL. These data suggest that the serum level of hepcidin can help identify subgroups of microsatellite stable metastatic colorectal cancer patients with a more aggressive course.

## 1. Introduction

Colorectal cancer (CRC) remains one of the highest-ranking cancers both in terms of incidence and mortality [1]. Despite effective screening programs, nearly one-fifth of CRC patients have distant metastasis at the time of diagnosis, and up to half of patients with initially localized disease will eventually develop metastatic progression [2]. In the last decade, the use of immune checkpoint inhibitors (ICIs) has largely improved the management of metastatic CRC (mCRC) patients with microsatellite instability [3]. In contrast, ICIs remain ineffective in microsatellite stable (MSS) mCRC patients, who represent approximately 90% of all advanced CRC cases [4,5]. Therefore, the 5-year overall survival (OS) rate of MSS mCRC patients remains lower than 20% after standard chemotherapy with or without targeted drugs [6]. These findings highlight the need for more effective treatments in the MSS mCRC population, as well as the identification of more reliable prognostic markers. Such markers could help identify patients with more aggressive tumor biology and resistance to standard first-line treatment regimens, who may be suitable candidates for experimental therapies within clinical trials.

Hepcidin is a well-known iron balance regulator, physiologically secreted by the liver [7]. Recent studies have shown that hepcidin can be also synthesized by CRC cells and functional studies have provided convincing evidence that CRC cell-derived hepcidin can target both cancer cells and tumor-infiltrating immune cells with the downstream effect of sustaining CRC cell growth and metastasis [8,9,10,11]. Furthermore, the evaluation of hepcidin production in the evolutive phases of CRC showed that the expression of hepcidin was greater in mCRC than in localized CRC, and survival analysis revealed that patients with the highest levels of hepcidin had a poor prognosis [8].

This study aimed to assess whether hepcidin was detectable in the serum of patients with mCRC and whether the baseline serum levels of hepcidin could serve as a prognostic biomarker in MSS mCRC patients eligible for first-line treatment with either FOLFOX-panitumumab or FOLFOX-bevacizumab, depending on the RAS or BRAF gene status.

## 2. Materials and Methods

### 2.1. Patients and Controls

This study was approved by the local Ethics Committee (protocol number 129/17) and included patients who were >18 years old with a diagnosis of either synchronous or metachronous mCRC. Demographic data (including age, sex, body mass index), common laboratory hematological, liver and kidney function tests, imaging, and histopathological findings (including N- and M-staging) were recorded.

Recorded variables were analyzed for their association with the primary outcome measure, which was overall survival (OS), calculated as the time from the day of serum sample collection to the date of death from any cause or last follow-up.

All the enrolled patients were eligible for first-line treatment with either FOLFOX- panitumumab (RAS/BRAF wild-type) or FOLFOX-bevacizumab (K-RAS or BRAF mutations). The controls included healthy volunteers with no recent history of gastrointestinal symptoms/signs and no familiarity of CRC. All patients signed the informed consent for this study. The research was carried out under The Code of Ethics of the World Medical Association (Declaration of Helsinki). Each patient who took part in this study gave written informed consent and the study protocol was approved by the local Ethics Committee (Tor Vergata University Hospital, Rome (129/17)).

### 2.2. Enzyme-Linked Immunosorbent Assay

Serum samples were prospectively collected from the controls and patients within one week before starting the planned pharmacological therapy. Serum hepcidin content was measured using a commercially available competitive enzyme-linked immunosorbent assay (ELISA) kit (R&D Systems, Minneapolis, MN, USA; assay range: 15.6–1000 pg/mL) according to the manufacturer’s instructions. Absorbance readings were taken at 450 nm using a multimode detector DTX 880 (Beckman Coulter, Milan, Italy).

### 2.3. Statistical Analysis

Normally distributed quantitative data are expressed as the mean ± standard deviation and were analyzed using Student’s *t*-test, while non-normally distributed data are expressed as the median (interquartile range) and were analyzed using the Mann–Whitney U-test. The cut-point level of serum hepcidin concentration providing the best separation of the OS rates (the primary outcome measure) into two groups was established using maximally selected rank statistics [11]. The OS was estimated using the Kaplan–Meier method, and groups were compared using the log-rank test. The multivariate Cox regression analysis was used to identify independent prognostic factors of OS, and the results of the analysis were expressed using hazard ratios (HRs) and 95% confidence intervals (CIs) with *p*-values. The following covariates were considered potentially independent prognostic factors and included in the multivariate Cox regression analysis: serum hepcidin levels (<40 vs. ≥40 ng/mL), CEA status, Karnofsky performance score (KPS) (<80 vs. ≥80), number of metastatic sites (<2 vs. ≥2), RAS/BRAF mutations (wild-type vs. mutations). The Spearman rank correlation coefficient was used to ascertain correlations between serum hepcidin levels and iron metabolism-related proteins.

A subgroup analysis for OS was also performed, accounting for the major clinically relevant classes, to investigate significant hepcidin/subgroup interaction and is presented as a forest plot with interacting *p*-values.

Because of the observational exploratory nature of the present prospective study, a formal calculation of the sample size was not mandatory. However, all consecutive patients meeting the inclusion criteria were enrolled, and the target sample size was set at about 50.

A *p*-value of less than 0.05 was considered statistically significant. All analyses were carried out with R (version 4.0.3).

## 3. Results

### 3.1. Characteristics of the Study Participants

Between January 2022 and December 2023, 55 patients with MSS mCRC (17 females; median age: 67 years, range: 41–72), five patients with non-mCRC (2 females, median age: 63, range: 53–73) and 35 healthy volunteers (normal controls) (21 females; median age: 45 years, range: 25–55) were prospectively enrolled. Serum samples were taken from each patient and control and analyzed for their content of hepcidin by ELISA. Among the mCRC patients, seven patients had rectal cancer while the remaining had colon cancer (Table 1). Twenty-one patients had metachronous mCRC. Thirty-three patients carried a RAS/BRAF-mutated tumor and were treated with FOLFOX-bevacizumab, and 22 patients had a RAS/BRAF wild-type tumor and were treated with FOLFOX-panitumumab; 18 patients had two or more distant metastases while in the remaining, there was a single metastatic localization. The median duration of follow-up was 10.4 months (95% CI: 6.1–15.2 months) based on the reverse Kaplan–Meier method.

### 3.2. Serum Hepcidin Levels Are Higher in mCRC Patients than in the Controls

**Figure 1:**
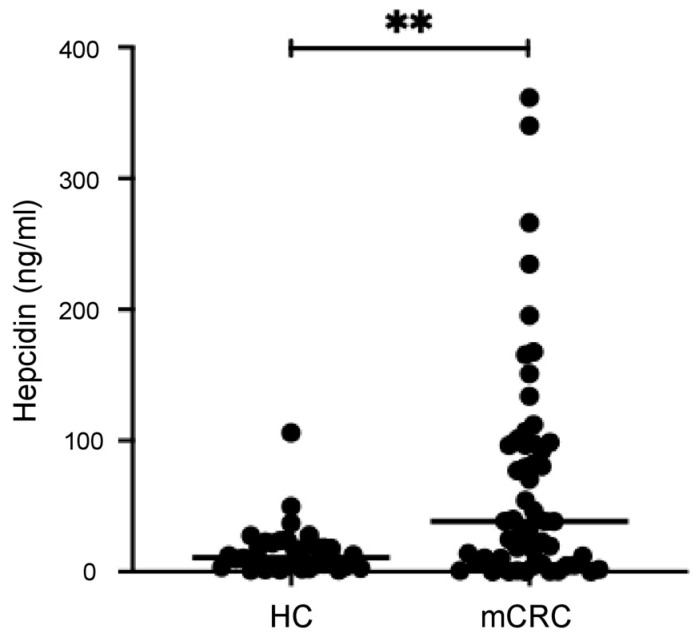
Serum hepcidin levels in healthy controls and patients with metastatic colorectal cancer (mCRC) as measured by ELISA. Each point in the graph indicates the value of serum hepcidin in a single control or single patient. ** *p* < 0.01.

Hepcidin was detectable in the serum samples of 52/55 (94.5%) mCRC patients and all controls. The median level of hepcidin was significantly higher in the serum samples of mCRC patients (38.38 ng/mL, range: 0–361.5) than in the controls (11 ng/mL, range: 0.97–106) (Figure 1, *p* < 0.0001). As an exploratory analysis, we also tested five patients with localized non-mCRC candidates for radical surgery. In these patients, the serum hepcidin levels (median: 18.49 ng/mL, range: 0–75.11) were greater than those in the controls, but the difference was not statistically significant.

When mCRC patients were stratified according to specific pathological/clinical features, it was evident that among patients with rectal localization of the disease, the fraction of those with high hepcidin levels was significantly greater than those with low hepcidin levels (Table 1). In contrast, there was no significant difference in terms of serum hepcidin levels between patients with synchronous or metachronous CRC and those with a single metastatic localization and two or more metastases (Table 1). Similarly, the serum hepcidin levels were not influenced by the RAS/BRAF status (Table 1).

### 3.3. Serum Hepcidin Level Is a Predictive Factor of the Overall Survival in mCRC

**Figure 2:**
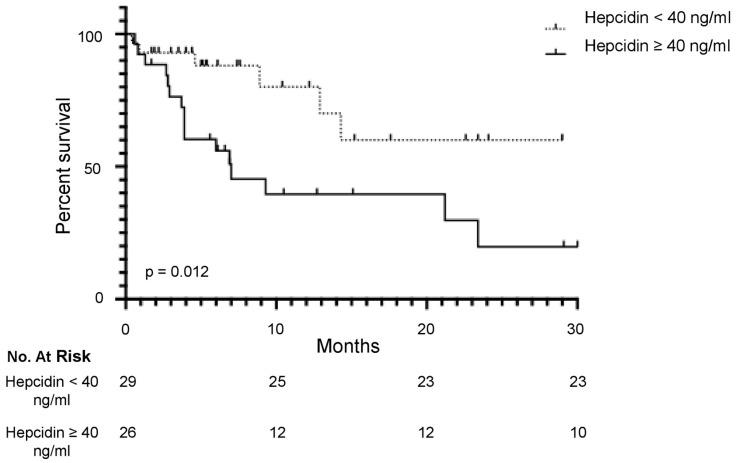
Percent survival at the specified time-points in patients with microsatellite stable (MSS) metastatic CRC (mCRC) treated with first-line treatment FOLFOX-panitumumab (RAS/BRAF wild-type) or FOLFOX-bevacizumab (RAS or BRAF mutations) and stratified by pre-treatment serum hepcidin levels. Patients were divided into two groups: the solid line indicates the high-hepcidin group (≥40 ng/mL), and the broken line indicates the low-hepcidin group (<40 ng/mL).

The median OS in the whole cohort was 14.3 months (95% CI: 7.0–23.4). To establish the optimal cut-point of hepcidin level by dividing patients into two groups with distinct survivals, we adopted maximally selected rank statistics. According to this analysis, the optimal cut-point level was 40 ng/mL. Of the 55 mCRC patients, 29 (52.7%) and 26 (47.2%) had values greater or lower than 40 ng/mL, respectively. Six out of 29 patients (20.6%) in the group with serum hepcidin levels lower than 40 ng/mL and 10/26 (38.4%) in the group with serum hepcidin levels greater than 40 ng/mL died during the follow-up. Consistently, patients with values of serum hepcidin greater than 40 ng/mL had a significantly shorter 1-year OS (39%) than those with hepcidin levels lower than 40 ng/mL (80%) (HR: 2.94; 95% CI: 1.27–6.84; *p* = 0.01) (Figure 2).

**Figure 3:**
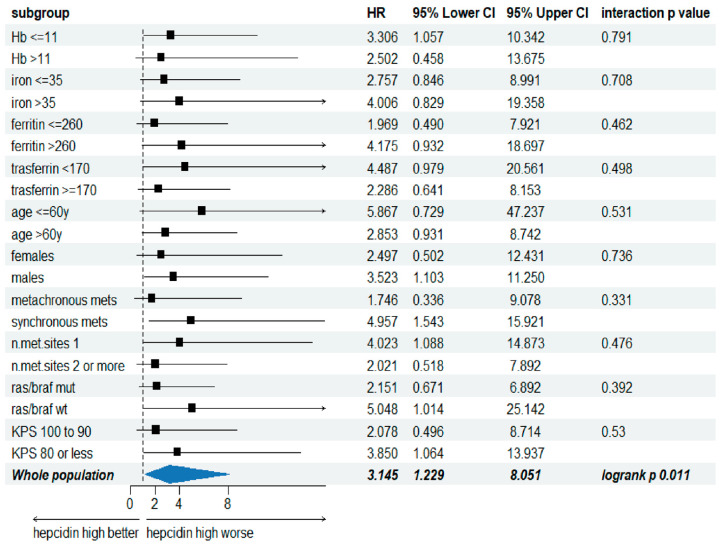
Overall survival for the specified clinically relevant patient subgroups (met = metastasis; KPS: Karnofsky performance score).

Next, we performed a subgroup analysis to evaluate whether the effect of serum hepcidin level on OS was consistent in all major clinically relevant patient subgroups, such as age, gender, hemoglobin, iron, ferritin and transferrin concentrations, timing of metastasis appearance, number of metastatic sites, K-RAS/BRAF mutation, and KPS. The OS effect of hepcidin was consistent in all analyzed subgroups, with a *p* for interaction ranging from 0.33 to 0.79 (Figure 3).

A multivariate Cox regression analysis including other well-known prognostic factors for mCRC (i.e., CEA, KPS, number of metastatic sites, K-RAS/BRAF mutation) showed that both the KPS (HR: 2.95, 95% CI: 1.12–7.81; *p* = 0.03) and serum hepcidin level greater than 40 ng/mL (HR: 2.68; 95% CI: 1.02–7.06; *p* = 0.04) were independent prognostic factors (Table 2).

A multivariate Cox regression analysis was also carried out including the pathologic stage of the primary tumor when resected (Stages I–II for 18 patients, Stage III for 15 patients, primary tumor not resected for 22 patients). Also in this multivariate analysis, the prognostic effect of hepcidin was retained (HR: 3.08; 95% CI: 1.20–7.90; *p* = 0.02). Staging was not associated with OS (using patients with primary not resected as a reference, Stages I–II and III *p*-values of 0.78 and 0.68, respectively).

### 3.4. Correlation Between Serum Hepcidin and Iron Metabolism-Related Factors

Hepcidin is a regulator of iron metabolism, and previous studies in other pathologies documented a positive correlation between serum hepcidin levels and serum ferritin levels [12]. In line with such data, we documented a mildly positive correlation between serum hepcidin and ferritin concentration (rho: 0.40; *p* = 0.002) while serum hepcidin levels did not correlate with hemoglobin, iron and transferrin concentrations (Table 3). To exclude that the prognostic effect of hepcidin was influenced by ferritin concentration, a multivariate Cox regression analysis was conducted with the two factors. The effect of serum hepcidin level on OS was retained (*p* = 0.0343; 95% IC: 1.08–7.47), while there was no significant prognostic effect of ferritin concentration on the OS (*p* = 0.4; 95% IC: 0.58–3.5).

## 4. Discussion

We and other authors recently showed that hepcidin is overproduced by CRC cells, particularly in the advanced stages of tumorigenesis [8]. By using the web server GEPIA database, we also documented that CRC patients with the highest tissue levels of hepcidin RNA have a reduced OS and disease-free survival as compared with patients with reduced levels of hepcidin transcripts [8]. Therefore, in this study, we explored the possibility that serum levels of hepcidin could help predict the prognosis of mCRC. For this purpose, we prospectively collected serum samples of patients with MSS mCRC eligible for first-line treatment with either FOLFOX-panitumumab (RAS/BRAF wild-type) or FOLFOX-bevacizumab (RAS or BRAF mutations) and analyzed the levels of hepcidin. Initially, we showed that hepcidin was detectable in almost all mCRC samples and normal samples, and the median level of hepcidin was significantly greater in mCRC serum samples than in the normal controls. Next, by adopting maximally selected rank statistics, we identified the value of serum hepcidin of 40 ng/mL as the best cut-point to divide the mCRC patients into two groups, with the most significant statistics between each other in terms of OS. Patients with baseline serum levels of hepcidin greater than 40 ng/mL had a significantly shorter 1-year OS than those with hepcidin levels lower than 40 ng/mL. A multivariate Cox regression analysis showed that the baseline serum hepcidin level was an independent prognostic factor for OS, and this was evident in all the major clinically relevant patient subgroups.

Hepcidin is mainly produced by hepatocytes, where various factors, including inflammatory cytokines and iron, regulate such synthesis positively [13,14]. Because both ferritin and hepcidin are upregulated by systemic iron overload, levels of hepcidin well correlate with those of ferritin in the serum [15]. Indeed, we found a positive correlation between serum hepcidin and ferritin levels. The prognostic value of serum ferritin levels has been investigated in patients with CRC, but the available evidence would seem to indicate that serum ferritin levels do not significantly influence cancer-related outcomes [16,17]. Nonetheless, to exclude the possibility that the effect of serum hepcidin level on OS in mCRC was somewhat influenced by the serum ferritin concentration, we performed a multivariate analysis and confirmed the prognostic role of serum hepcidin level on OS.

Our findings confirm and expand previously published data documenting high levels of serum hepcidin in other advanced cancers. For instance, serum hepcidin levels are increased in patients with acute leukemia (AL) compared to the normal controls and predict worse outcomes in AL patients undergoing hematopoietic cell transplantation [18]. Similarly, high serum hepcidin levels have been documented in patients with breast cancer as compared to normal subjects and patients with benign breast diseases [19] as well as in patients with metastatic gastric cancer, patients with non-small cell lung cancer and patients with multiple myeloma [20,21,22]. Elevated serum hepcidin levels predict the aggressiveness and progression of renal cell carcinoma and upper urinary tract urothelial carcinomas [23,24]. In this context, it is, however, noteworthy that the upregulation of the circulating levels of hepcidin is not a hallmark of all cancers because the serum levels of hepcidin are reduced in patients with nasopharyngeal carcinoma as well as in patients with hepatocellular carcinoma [25,26,27].

Our study had some limitations. It was conducted on a small sample of patients who were followed for a relatively short period. This did not make it possible to collect data on additional endpoints other than OS (e.g., relapse incidence). However, the prospective nature of this study and detailed characterization of the mCRC patients allowed us to draw some relevant pieces of information. This study was not designed to ascertain the cell source of the circulating levels of hepcidin in mCRC. However, the fact that hepcidin is produced by CRC cells, mainly in the advanced stages of the disease [8], raises the possibility that the levels of the hormone measured in the serum samples were a spillover of the CRC-derived hepcidin. The controls of this study were slightly younger than the mCRC patients, but it is unlikely that this could have influenced the results because previous studies showed that serum hepcidin levels were stable over time in the general population [15].

## 5. Conclusions

The present findings show that the circulating levels of hepcidin greater than 40 ng/mL are an independent risk factor for OS in MSS mCRC patients undergoing standard first-line treatment. Further prospective and extensive studies are needed to confirm and validate our findings, as well as to standardize serum hepcidin evaluation and ascertain whether patients with the highest serum levels of hepcidin could benefit from other therapies.

## Figures and Tables

**Table 1:** Demographic, clinical and laboratory characteristics of enrolled patients.

Characteristics	Hepcidin ≥ 40 ng/mL (N = 26)	Hepcidin < 40 ng/mL (N = 29)	*p*-Value
Age (years), median [range]	63 (44–91)	70 (41–85)	0.1085
Female gender, n (%)	10 (38.5)	7 (24)	0.4000
Disease localization, n (%):			
Rectal	5 (19.2)	2 (7)	0.0057
Colon	21 (80.8)	27 (93)	
Metastasis, n (%):			
1	16 (61.5)	21 (72.4)	0.7289
>1	10 (38.5)	8 (27.6)	
Sync, n (%):	16 (61.5)	18 (62)	
Meta, n (%):	10 (38.5)	11 (38)	0.5883
RAS/BRAF, n (%):			
WT	9 (34.6)	13 (44.8)	0.3982
MUT	17 (65.4)	16 (55.2)	
BMI, n (%):			
>21.7	14 (54)	25 (86)	0.2118
≤21.7	12(46)	4 (14)	
KPS, n (%):			
<80	13 (50)	10 (34.5)	0.3213
90–100	13 (50)	19 (65.5)	
CEA, n (%):			
>1.82	20 (77)	15 (52)	0.1694
≤1.82	6 (23)	14 (48)	
Hb, n (%):			
>11.5	10 (38.5)	15 (52)	0.8969
≤11.5	16 (61.5)	14 (48)	

**Table 2:** Multivariate analysis of the overall survival. CI: confidence interval; KPS: Karnofsky performance score.

Variable	*p*	Hazard Ratio	95% CI
CEA	0.1370	1.0009	0.9997–1.0020
Hepcidin > 40 ng/mL	0.0460	2.6796	1.0175–7.0569
kPS ≤ 80	0.0291	2.9528	1.1168–7.8073
Number of metastatic sites ≥2	0.7149	1.1984	0.4538–3.1648
RAS/BRAF wild-type	0.8760	1.0786	0.4171–2.7895

**Table 3:** Correlation between serum hepcidin levels and iron metabolism-related factors.

		Ferritin Concentration (ng/mL)	Iron Concentration (μg/dL)	Transferrin Concentration (mg/dL)	Hemoglobin Concentration (g/dL)
Iron	Correlation	0.146			
Concentration	Coefficient				
(μg/dL)					
	*p*	0.2888			
	n	55			
Transferrin	Correlation	−0.408	0.129		
Concentration	Coefficient				
(mg/dL)					
	*p*	0.0020	0.3469		
	n	55	55		
Hemoglobin	Correlation	0.022	0.264	−0.174	
Concentration	Coefficient				
(g/dL)					
	*p*	0.8736	0.0513	0.2033	
	n	55	55	55	
Hepcidin	Correlation	0.399	0.159	−0.207	−0.126
Level (ng/mL)	Coefficient				
	*p*	0.0025	0.2469	0.1287	0.3577
	n	55	55	55	55

## Data Availability

The data included in this study are available from the corresponding authors upon reasonable request.
